# Supplementary material for: Impact of early corticosteroids on 60-day mortality in critically ill patients with COVID-19: A multicenter cohort study of the OUTCOMEREA network
Source: PLoS One. 2021 Aug 4;16(8):e0255644. doi: 10.1371/journal.pone.0255644 (PMC8336847; doi:10.1371/journal.pone.0255644)
Supplement: S2 Fig — ICU: Intensive care unit; SOFA: Sequential Organ Failure Assessment; AUC: 0.76. (DOCX) [file pone.0255644.s002.docx]

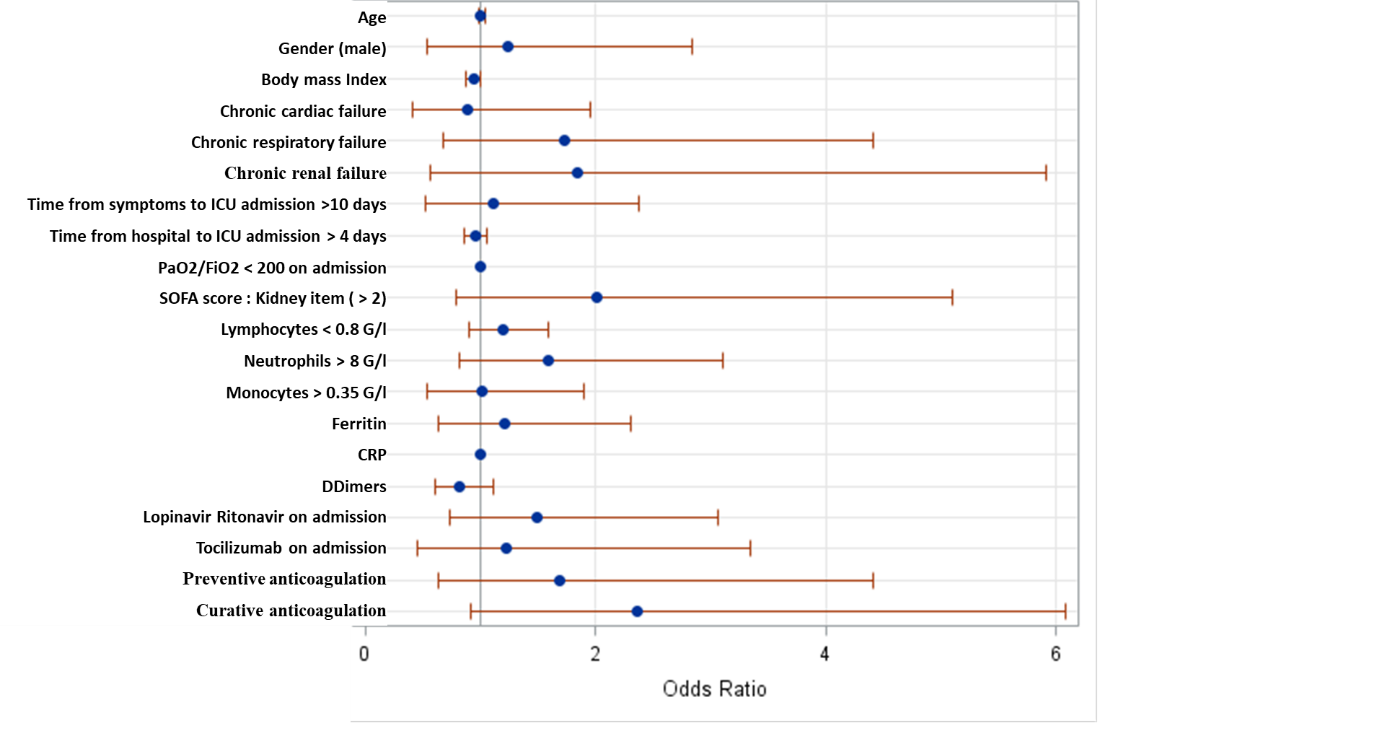


**S2** **Fig Propensity score: multivariate regression logistic analysis for the factors associated with receiving early Corticosteroids.**

ICU: Intensive care unit; SOFA: Sequential Organ Failure Assessment

AUC=0.73
