# Supplementary material for: Impact of early corticosteroids on 60-day mortality in critically ill patients with COVID-19: A multicenter cohort study of the OUTCOMEREA network
Source: PLoS One. 2021 Aug 4;16(8):e0255644. doi: 10.1371/journal.pone.0255644 (PMC8336847; doi:10.1371/journal.pone.0255644)
Supplement: S3 Fig — * a Ferritin > 1000 μg/l or D-Dimers > 1000 μg/l or C-Reactive Protein > 100 mg/dL. (DOCX) [file pone.0255644.s003.docx]

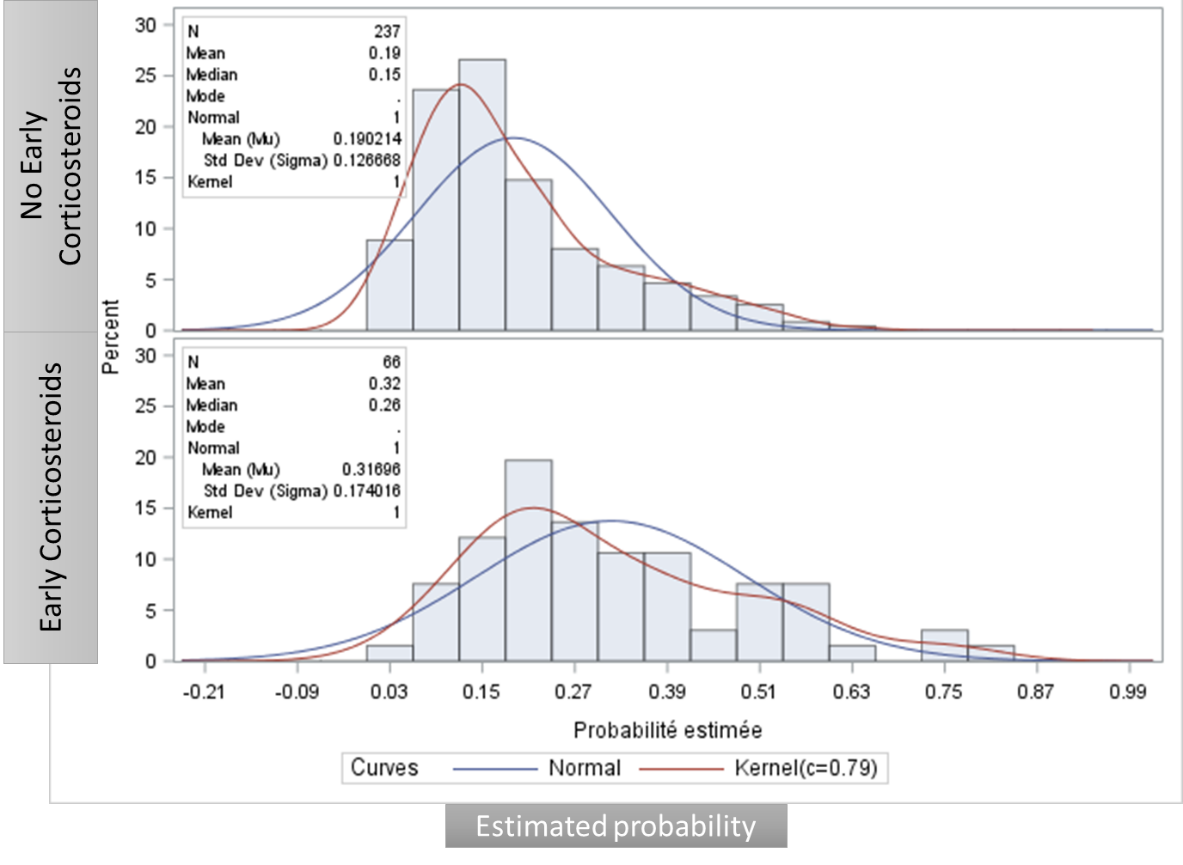


**S3 Fig: Histograms of propensity score by treatment group: Early Corticosteroids and Non-early Corticosteroids**
