# Supplementary material for: Impact of early corticosteroids on 60-day mortality in critically ill patients with COVID-19: A multicenter cohort study of the OUTCOMEREA network
Source: PLoS One. 2021 Aug 4;16(8):e0255644. doi: 10.1371/journal.pone.0255644 (PMC8336847; doi:10.1371/journal.pone.0255644)
Supplement: S4 Fig — Standardized differences in the covariates included in the propensity score, before (in blue) and after (in red) weighting. (DOCX) [file pone.0255644.s004.docx]

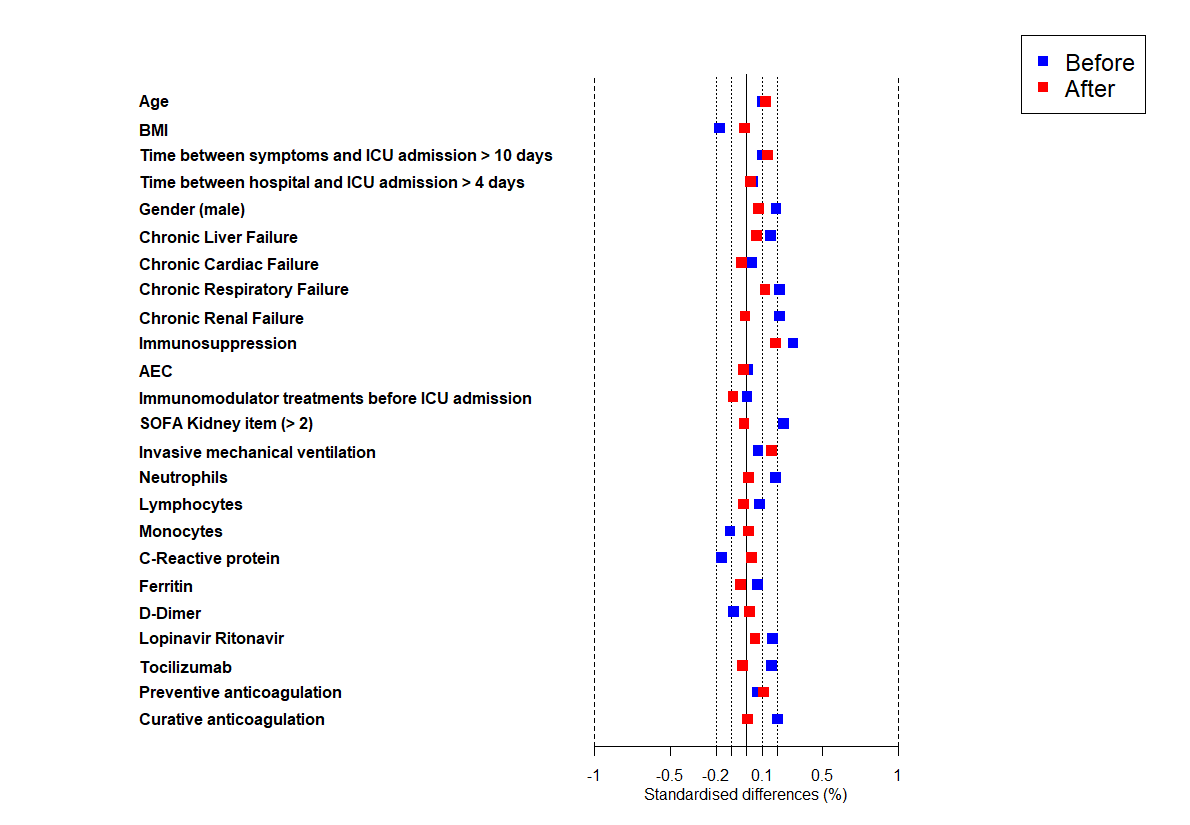


**S4 Fig: Standardized differences in the covariates included in the propensity score, before (in blue) and after (in red) weighting.**
