# Supplementary material for: Impact of early corticosteroids on 60-day mortality in critically ill patients with COVID-19: A multicenter cohort study of the OUTCOMEREA network
Source: PLoS One. 2021 Aug 4;16(8):e0255644. doi: 10.1371/journal.pone.0255644 (PMC8336847; doi:10.1371/journal.pone.0255644)
Supplement: S6 Fig — (DOCX) [file pone.0255644.s006.docx]

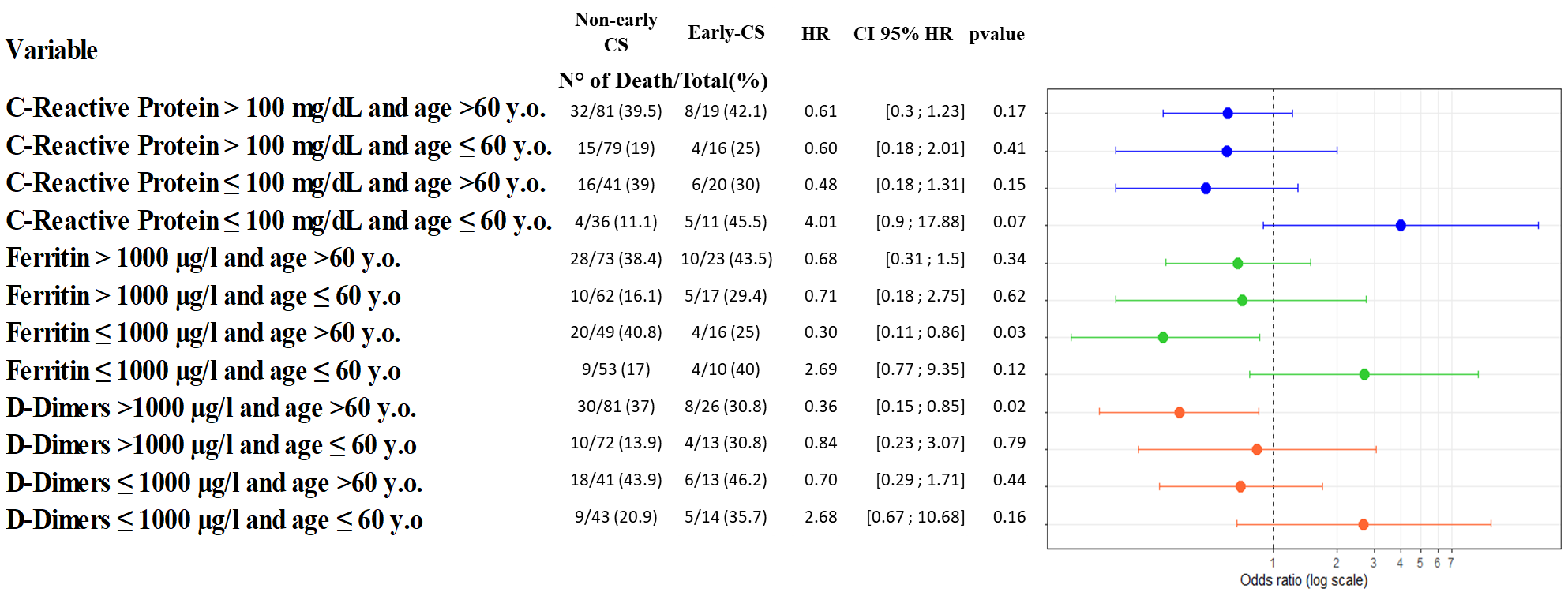


**S6 Figure: Subgroup analyses based on C-Reactive protein, Ferritin, and D-Dimer levels and interaction with age.**
