# Supplementary material for: Impact of early corticosteroids on 60-day mortality in critically ill patients with COVID-19: A multicenter cohort study of the OUTCOMEREA network
Source: PLoS One. 2021 Aug 4;16(8):e0255644. doi: 10.1371/journal.pone.0255644 (PMC8336847; doi:10.1371/journal.pone.0255644)
Supplement: S1 Table. Cox model for 60-day mortality with ponderation on IPTW for the impact of early corticosteroids. IPTW: Inverse Probability of treatment weight; HR: Hazard Ratio — (DOCX) [file pone.0255644.s007.docx]

**S1 Table: Cox model for 60-day mortality with ponderation on IPTW for the impact of early corticosteroids**

| Truncation | Estimated weight | | Early corticosteroids effect | | |
| --- | --- | --- | --- | --- | --- |
| Percentiles | Mean (SD) | Minimum/maxium | HR | CI 95% | P-value |
| 0,1 | 1/1.28 | 0.495/13.503 | 0.80 | [0.51 ; 1.26] | 0.33 |
| 1,99 | 0.998/1.279 | 0.497/13.503 | 0.81 | [0.51 ; 1.27] | 0.36 |
| 5 ,95 | 0.946/0.936 | 0.506/6.438 | 0.86 | [0.54 ; 1.35] | 0.51 |
| 10,9 | 0.91/0.769 | 0.514/4.363 | 0.87 | [0.54 ; 1.39] | 0.56 |
| 25,75 | 0.84/0.525 | 0.539/2.44 | 0.96 | [0.59 ; 1.57] | 0.88 |
| 50,50 | 0.789/0.377 | 0.59/1.502 | 1.06 | [0.64 ; 1.77] | 0.81 |

IPTW: Inverse Probability of treatment weight; HR: Hazard Ratio
