# Supplementary material for: Impact of early corticosteroids on 60-day mortality in critically ill patients with COVID-19: A multicenter cohort study of the OUTCOMEREA network
Source: PLoS One. 2021 Aug 4;16(8):e0255644. doi: 10.1371/journal.pone.0255644 (PMC8336847; doi:10.1371/journal.pone.0255644)
Supplement: S2 Table — HR: Hazard Ratio; SOFA: Sequential Organ Failure Assessment; HR: Hazard Ration. (DOCX) [file pone.0255644.s008.docx]

**S2 Table: Multivariate survival analyses of the factors associated with 60-day mortality.**

| Variables | HR | HRIC | Pr > Khi-2 |
| --- | --- | --- | --- |
| Early corticosteroids | 1.10 | [0.63 ; 1.92] | 0.74 |
| Time between symptoms and ICU admission | 0.66 | [0.41 ; 1.07] | 0.09 |
| Chronic cardiac disease | 1.93 | [1.19 ; 3.12] | 0.01 |
| Chronic respiratory disease | 2.11 | [1.14 ; 3.89] | 0.02 |
| PaO2/FiO2 < 200 mmHg | 1.93 | [1.15 ; 3.23] | 0.01 |
| Vasopressors | 1.65 | [1.08 ; 2.54] | 0.02 |
| Age | 1.06 | [1.04 ; 1.09] | <0.01 |
| Renal SOFA > 2 | 1.76 | [0.95 ; 3.25] | 0.07 |
| Leucocytes > 10 G/L | 1.44 | [0.92 ; 2.25] | 0.11 |
| Monocytes > 1 G/L | 0.66 | [0.42 ; 1.03] | 0.07 |
| T° > 39 °C | 2.06 | [1.24 ; 3.45] | <0.01 |

HR: Hazard Ratio; SOFA: Sequential Organ Failure Assessment; HR: Hazard Ration
