# Supplementary material for: Impact of early corticosteroids on 60-day mortality in critically ill patients with COVID-19: A multicenter cohort study of the OUTCOMEREA network
Source: PLoS One. 2021 Aug 4;16(8):e0255644. doi: 10.1371/journal.pone.0255644 (PMC8336847; doi:10.1371/journal.pone.0255644)
Supplement: S3 Table — Inflammation* Ferritin >1000 μg/l or D-Dimers >1000 μg/l or C-Reactive Protein >100 mg/dL; VFD: Ventilatory free days; BSI: Blood stream infection, HAP-VAP: hospital-acquired pneumonia and ventilator-associated pneumonia. LOS: Length of stay; HSHC: Hydrocortisone hemisuccinate ICU: intensive care unit; SOFA: Sequential organ Failure assessment; SAPS II: simplified acute physiology score. (DOCX) [file pone.0255644.s009.docx]

**S3 Table: Comparison between older and younger patients and between patients with and without inflammation**

|  | Age < 60 y.o. | Age > 60 y.o. | pvalue | No inflammation | Inflammation | pvalue |
| --- | --- | --- | --- | --- | --- | --- |
| **Number of patients** | 142 | 161 |  | 101 (100) | 202 (100) | . |
| **Baseline characteristics** |  |  |  |  |  |  |
| Age | 52 [46 ; 57] | 70 [65 ; 74] | <0.01 | 60 [52 ; 70] | 62 [54 ; 70] | 0.92 |
| Body-mass index, kg/cm² * (miss=14) | 29.1 [25.9 ; 33.1] | 27.7 [25.2 ; 30.7] | <0.01 | 28.4 [25.9 ; 32.2] | 28.2 [25.2 ; 31.3] | 0.19 |
| Body-mass index ≥ 30 | 63 (44.4) | 45 (28) | <0.01 | 43 (42.6) | 64 (31.7) | 0.06 |
| Comorbidities |  |  |  |  |  |  |
| At least one comorbidity | 67 (47.2) | 109 (67.7) | <0.01 | 62 (61.4) | 114 (56.4) | 0.41 |
| Chronic liver failure | 3 (2.1) | 1 (0.6) | 0.26 | 2 (2) | 2 (1) | 0.48 |
| Chronic cardiovascular disease | 27 (19) | 52 (32.3) | <0.01 | 21 (20.8) | 58 (28.7) | 0.14 |
| Chronic respiratory failure | 16 (11.3) | 17 (10.6) | 0.84 | 9 (8.9) | 24 (11.9) | 0.43 |
| Chronic kidney disease | 8 (5.6) | 14 (8.7) | 0.31 | 9 (8.9) | 13 (6.4) | 0.43 |
| Immunosuppression§ | 6 (4.2) | 5 (3.1) | 0.60 | 4 (4) | 7 (3.5) | 0.83 |
| Time between symptoms and ICU admission | 10 [8 ; 12] | 10 [7 ; 12] | 0.45 | 10 [8 ; 12] | 9.5 [7 ; 12] | 0.55 |
| **Treatment before admission** |  |  |  |  |  |  |
| Angiotensin converting enzyme inhibitor | 18 (12.7) | 41 (25.5) | <0.01 | 25 (24.8) | 34 (16.8) | 0.10 |
| Immunomodulatory treatments | 7 (4.9) | 2 (1.2) | 0.06 | 8 (7.9) | 1 (0.5) | <.01 |
| **Characteristics on admission** |  |  |  |  |  |  |
| SAPS II score | 28 [22 ; 36] | 36 [30 ; 47] | <0.01 | 33 [25.5 ; 42] | 32 [25 ; 45] | 0.51 |
| SOFA score | 4 [2 ; 6] | 4 [3 ; 7] | 0.07 | 4 [2 ; 6] | 4 [3 ; 7] | 0.93 |
| Neurological failure (GCS < 15) | 21 (14.8) | 19 (11.8) | 0.44 | 12 (11.9) | 28 (13.9) | 0.63 |
| Body temperature > 39°C | 58 (40.8) | 38 (23.6) | <0.01 | 21 (20.8) | 75 (37.1) | <.01 |
| **Severity of ARDS** |  |  |  |  |  |  |
| No ARDS PaO2/FiO2 > 300 | 16 (11.3) | 21 (13) | 0.54 | 13 (12.9) | 24 (11.9) | 0.88 |
| Mild: PaO2/FiO2 200-300 | 18 (12.7) | 26 (16.1) | . | 13 (12.9) | 31 (15.3) | . |
| Moderate: PaO2/FiO2  100-200 | 61 (43) | 72 (44.7) | . | 43 (42.6) | 90 (44.6) | . |
| Severe: PaO2/FiO2 < 100 | 47 (33.1) | 42 (26.1) | . | 32 (31.7) | 57 (28.2) | . |
| **Ventilatory support on admission** |  |  |  |  |  |  |
| No mechanical ventilation | 59 (41.5) | 65 (40.4) | 0.20 | 40 (39.6) | 84 (41.6) | 0.24 |
| Non-invasive mechanical ventilation | 28 (19.7) | 45 (28) | . | 30 (29.7) | 43 (21.3) | . |
| Mechanical ventilation | 55 (38.7) | 51 (31.7) | . | 31 (30.7) | 75 (37.1) | . |
| **Laboratory features on admission** |  |  |  |  |  |  |
| Leucocytes (miss=10) | 7605 [5400 ; 10680] | 7900 [6000 ; 10500] | 1.00 | 6900 [4800 ; 9700] | 8250 [6400 ; 11751.8] | <.01 |
| Neutrophils (miss=36) | 6650 [4300 ; 9830] | 6710 [4980 ; 9239.5] | 0.96 | 6040 [3780 ; 9460] | 7400 [5400 ; 11100] | <.01 |
| Lymphocytes (miss=36) | 900 [630 ; 1210] | 700 [500 ; 1070.7] | <0.01 | 780 [500 ; 1000] | 845 [600 ; 1260] | 0.05 |
| Monocytes(miss=36) | 340 [201.1 ; 500] | 390 [228.2 ; 600] | 0.53 | 310 [200 ; 500] | 400 [230 ; 600] | 0.12 |
| CRP(miss=63) | 139.7 [84 ; 213] | 168 [83.5 ; 238] | 0.13 | 95 [60 ; 162] | 181.4 [126 ; 252] | <.01 |
| Ferritin (miss=107) | 1135.5 [491 ; 1960.2] | 974 [622 ; 1801] | 0.76 | 727 [355 ; 1044] | 1355.6 [723 ; 2302] | <.01 |
| D-Dimers (miss=90) | 1626.5 [800 ; 3957.3] | 2349.4 [980 ; 6500] | 0.02 | 851 [600 ; 1800] | 2804.6 [1300 ; 6900] | <.01 |
| Inflammation* | 119 (83.8) | 135 (83.9) | 0.99 |  |  |  |
| **Treatments on admission** |  |  |  |  |  |  |
| Lopinavir Ritonavir | 44 (31) | 65 (40.4) | 0.09 | 34 (33.7) | 75 (37.1) | 0.55 |
| Tocilizumab | 13 (9.2) | 12 (7.5) | 0.59 | 15 (14.9) | 10 (5) | <.01 |
| Anakinra | 11 (7.7) | 11 (6.8) | 0.76 | 5 (5) | 17 (8.4) | 0.27 |
| Hydroxychloroquine | 20 (14.1) | 13 (8.1) | 0.09 | 13 (12.9) | 20 (9.9) | 0.43 |
| Corticosteroids | 27 (19) | 39 (24.2) | 0.27 | 23 (22.8) | 43 (21.3) | 0.77 |
| High dose of corticosteroids | 25 (17.6) | 30 (18.6) | 0.82 | 17 (16.8) | 38 (18.8) | 0.67 |
| First corticosteroids after Day 3 | 44 (31) | 50 (31.1) | 0.99 | 46 (45.5) | 48 (23.8) | <.01 |
| **LOS and Mortality** |  |  |  |  |  |  |
| ICU LOS | 12 [7 ; 20] | 11 [7 ; 20] | 0.84 | 12 [7 ; 19] | 11.5 [7 ; 21] | 0.49 |
| ICU Death | 28 (19.7) | 58 (36) | <0.01 | 25 (24.8) | 61 (30.2) | 0.32 |
| Death at day 60 | 28 (19.7) | 62 (38.5) | <0.01 | 26 (25.7) | 64 (31.7) | 0.29 |
| **Adverse events due to corticosteroids** |  |  |  |  |  |  |
| VFD | 4 [1 ; 7] | 3 [1 ; 8] | 0.70 | 4 [2 ; 6] | 3 [1 ; 7] | 0.32 |
| Hyperglycemia | 53 (37.3) | 52 (32.3) | 0.36 | 39 (38.6) | 66 (32.7) | 0.31 |
| Mean daily dose of insulin | 8.5 [0 ; 38.8] | 9 [0 ; 49.9] | 0.45 | 10.8 [0 ; 44.2] | 7.1 [0 ; 44.1] | 0.20 |
| BSI | 22 (15.5) | 21 (13) | 0.54 | 8 (7.9) | 35 (17.3) | 0.03 |
| HCAP | 46 (32.4) | 49 (30.4) | 0.71 | 28 (27.7) | 67 (33.2) | 0.34 |

Inflammation* Ferritin >1000 µg/l or D-Dimers >1000 µg/l or C-Reactive Protein >100 mg/dL

VFD: Ventilatory free days; BSI: Blood stream infection, HAP-VAP: hospital-acquired pneumonia and ventilator-associated pneumonia. LOS: Length of stay; HSHC: Hydrocortisone hemisuccinate ICU: intensive care unit; SOFA: Sequential organ Failure assessment; SAPS II: simplified acute physiology score
