# Supplementary material for: Impact of early corticosteroids on 60-day mortality in critically ill patients with COVID-19: A multicenter cohort study of the OUTCOMEREA network
Source: PLoS One. 2021 Aug 4;16(8):e0255644. doi: 10.1371/journal.pone.0255644 (PMC8336847; doi:10.1371/journal.pone.0255644)
Supplement: S4 Table — HAP-VAP: Ventilator associated pneumoniae; SubHR: Sub hazard ratio. (DOCX) [file pone.0255644.s010.docx]

S4 Table: Complete case analyses

|  | Non-early CS | Early-CS | HR | CI 95% HR | pvalue |
| --- | --- | --- | --- | --- | --- |
|  | **N° of Death/Total(%)** | |  |  |  |
| Inflammation | 20/70 (28.6) | 12/33 (36.4) | 0.73 | [0.35 ; 1.55] | 0.41 |
| No inflammation | 16/66 (24.2) | 8/21 (38.1) | 1.30 | [0.55 ; 3.07] | 0.55 |
| Inflammation and age > 60 yo | 24/55 (43.6) | 9/23 (39.1) | 0.54 | [0.25 ; 1.18] | 0.12 |
| Inflammation and age ≤ 60 yo | 4/13 (30.8) | 2/4 (50) | 0.57 | [0.08 ; 4.02] | 0.57 |
| No inflammation and age > 60 yo | 5/58 (8.6) | 5/21 (23.8) | 0.92 | [0.23 ; 3.63] | 0.90 |
| No inflammation and age ≤ 60 yo | 3/10 (30) | 4/6 (66.7) | 4.45 | [0.78 ; 25.39] | 0.09 |
| C-reactive protein > 100 mg/dL | 36/134 (26.9) | 12/32 (37.5) | 0.93 | [0.49 ; 1.77] | 0.82 |
| C-Reactive Protein ≤ 100 mg/dL | 15/66 (22.7) | 10/26 (38.5) | 1.44 | [0.58 ; 3.59] | 0.43 |
| C-reactive protein > 100 mg/dL and age > 60 yo | 27/69 (39.1) | 8/16 (50) | 0.84 | [0.38 ; 1.85] | 0.66 |
| C-reactive protein > 100 mg/dL and age ≤ 60 yo | 9/65 (13.8) | 4/16 (25) | 0.78 | [0.21 ; 2.92] | 0.71 |
| C-Reactive Protein ≤ 100 mg/dL and Age > 60 yo | 11/33 (33.3) | 5/15 (33.3) | 0.43 | [0.13 ; 1.44] | 0.17 |
| C-Reactive Protein ≤ 100 mg/dL and Age ≤ 60 yo | 4/33 (12.1) | 5/11 (45.5) | 4.01 | [0.9 ; 17.88] | 0.07 |
| Ferritin > 1000 µg/L | 22/71 (31) | 13/31 (41.9) | 0.94 | [0.43 ; 2.05] | 0.87 |
| Ferritin ≤ 1000 µg/L | 19/83 (22.9) | 8/24 (33.3) | 1.02 | [0.47 ; 2.23] | 0.96 |
| Ferritin > 1000 µg/L and age > 60 yo | 16/38 (42.1) | 8/14 (57.1) | 0.72 | [0.24 ; 2.14] | 0.56 |
| Ferritin > 1000 µg/L and age ≤ 60 yo | 6/33 (18.2) | 5/17 (29.4) | 0.66 | [0.16 ; 2.72] | 0.57 |
| Ferritin ≤ 1000 µg/L and age > 60 yo | 15/40 (37.5) | 4/14 (28.6) | 0.39 | [0.13 ; 1.16] | 0.09 |
| Ferritin ≤ 1000 µg/L and Age ≤ 60 yo | 4/43 (9.3) | 4/10 (40) | 5.70 | [1.14 ; 28.51] | 0.03 |
| D-Dimers > 1000 µg/L | 18/90 (20) | 10/30 (33.3) | 0.84 | [0.37 ; 1.91] | 0.68 |
| D-Dimers ≤ 1000 µg/L | 22/71 (31) | 10/26 (38.5) | 1.22 | [0.58 ; 2.54] | 0.61 |
| D-Dimers > 1000 µg/L and age > 60 yo | 16/46 (34.8) | 6/17 (35.3) | 0.39 | [0.12 ; 1.25] | 0.11 |
| D-Dimers > 1000 µg/L and age ≤ 60 yo | 2/44 (4.5) | 4/13 (30.8) | 1.90 | [0.27 ; 13.54] | 0.52 |
| D-Dimers ≤ 1000 µg/L and age > 60 yo | 15/33 (45.5) | 5/12 (41.7) | 0.64 | [0.26 ; 1.61] | 0.34 |
| D-Dimers ≤ 1000 µg/L and Age ≤ 60 yo | 7/38 (18.4) | 5/14 (35.7) | 2.68 | [0.67 ; 10.68] | 0.16 |
