# Supplementary material for: Impact of early corticosteroids on 60-day mortality in critically ill patients with COVID-19: A multicenter cohort study of the OUTCOMEREA network
Source: PLoS One. 2021 Aug 4;16(8):e0255644. doi: 10.1371/journal.pone.0255644 (PMC8336847; doi:10.1371/journal.pone.0255644)
Supplement: S5 Table — ICU BSI: Intensive care unit blood stream infection; SubHR: Sub Hazard Ratio. (DOCX) [file pone.0255644.s011.docx]

S5 Table: Association between Ferritin, CRP, D-Dimers and the occurrence of death: area under the ROC curve

|  | AUC | threshold | specificity | sensitivity | accuracy |
| --- | --- | --- | --- | --- | --- |
| Ferritin | 0.57 [0.5 - 0.64] | 1816 | 0.75 | 0.41 | 0.65 |
| C-reactive Protein | 0.55 [0.47 - 0.62] | 187 | 0.68 | 0.42 | 0.6 |
| DDimers | 0.54 [0.47 - 0.61] | 1099.5 | 0.7 | 0.42 | 0.62 |

AUC: Area Under the Curve
